# *Bdellovibrio bacteriovorus*: mechanisms, problems, and prospects against MDR respiratory infections

**DOI:** 10.3389/fmicb.2026.1736645

**Published:** 2026-02-27

**Authors:** Ashir Kanwal, Bismah Azeem, Mohammed Hammad Jaber Amin, Leina Elomeiri

**Affiliations:** 1 Department of Internal Medicine, Liaquat University of Medical and Health Sciences, Jamshoro, Pakistan; 2 Department of Internal Medicine, Avicenna Medical College, Lahore, Pakistan; 3 Department of Internal Medicine, Alzaiem Alazhari University, Khartoum North, Sudan; 4 Department of Internal Medicine, University of Khartoum, Khartoum, Sudan

**Keywords:** *Bdellovibrio bacteriovorus*, biofilm, Gram-negative, living antibiotic, multidrug resistance, predatory bacteria, respiratory infections

## Abstract

The rising rate of multidrug resistance (MDR) among bacteria is a real threat to public health across the globe, necessitating novel solutions that are beyond pharmacological approaches. The appearance of *Bdellovibrio bacteriovorus* as a predatory Gram-negative bacterium has become an alternative source to treat antibiotic-resistant infections, especially in cases of respiratory tract infections by pathogenic organisms like *Klebsiella pneumoniae* and *Pseudomonas aeruginosa*. *In vivo* studies demonstrate that intranasal administration of *Bdellovibrio bacteriovorus* reduces *Klebsiella pneumoniae* lung burden by more than 3 log_10_ in rat models, while *in vitro* studies report significant disruption of *P. aeruginosa* biofilms and reduced epithelial invasion. The targeting of Gram-negative bacteria by this bacterium is the key to success, as this bacterium can prey upon Gram-negative bacteria and biofilm-forming bacteria. Nonetheless, Gram-positive bacteria have not been fully utilized in the application of *Bdellovibrio bacteriovorus*. In this review, our study aims to discuss antibiotic resistance, the *Bdellovibrio* life cycle, *Bdellovibrio bacteriovorus* against Gram-negative and Gram-positive bacteria, its shortcomings, and the way it could be applied, most especially against respiratory infections. However, important limitations remain, including incomplete eradication of prey populations, transient phenotypic resistance, and the absence of long-term or human clinical safety data. This review highlights the potential of *B. bacteriovorus* as an active antibacterial agent and draws attention to its clinical use to solve the current MDR respiratory infections.

## 1 Introduction

The mid-20th century marked the Golden Age of antibiotics; however, over time, pathogens evolved to become resistant, leading to multidrug-resistant bacteria (MDR) (Saralegui et al., 2022; Baquero, 2021), a major public health threat of the 21st century (Antimicrobial Resistance Collaborators, 2022). The World Health Organization (WHO) has identified several priority bacterial pathogens. They are *Staphylococcus aureus, Streptococcus pneumoniae*, both Gram-positive cocci, *Escherichia coli, Klebsiella* *pneumoniae, Acinetobacter baumannii, and Pseudomonas aeruginosa*, which are Gram-negative bacilli. These pathogens matter as far as resistance to antimicrobials and hospital-acquired infections are concerned, as major contributors to the Antimicrobial Resistance (AMR) burden in 2019 (Antimicrobial Resistance Collaborators, 2022; World Health Organization, 2017a). Antibiotics transformed medicine by effectively combating once-deadly bacterial infections, hence providing miraculous solutions (Estany-Gestal et al., 2024).

Lower respiratory tract infections (LRTIs) are very common among people, causing numerous deaths globally, with an estimated count of around 2.74 million deaths each year (Santella et al., 2021). The older population is more prone to LRTIs than young adults. At the same time, children hospitalized with LRTIs often suffer from severe illness, extended recovery, and potential respiratory complications affecting around a quarter of them (Duan et al., 2020). The most typical LRTIs include conditions like acute bronchitis, acute tracheobronchitis, chronic bronchitis, and pneumonia. These illnesses cause hospital admissions of about 4.4% and are linked to serious health problems, deaths, and increased healthcare expenses (Santella et al., 2021). Regions with low sociodemographic status, reliance on solid fuels for cooking and heating, as well as malnourished and immunocompromised populations, bear the greatest burden of lower respiratory tract infections (GBD 2015 LRI Collaborators, 2017).

The primary bacteria causing LRTIs include Gram-positive types such as *Staphylococcus aureus* and *Enterococcus species*, and Gram-negative types like *Pseudomonas species, Acinetobacter species, Klebsiella pneumoniae*, and *Haemophilus influenzae* (Santella et al., 2021). Colistin and Linezolid are useful against *Staphylococcus aureus* and *Enterococcus species*, whereas Colistin alone is commonly used against *Pseudomonas, Acinetobacter* species, *Klebsiella pneumoniae*, and *Haemophilus influenzae*; however, increasing resistance has been reported (Santella et al., 2021). The rising prevalence of extended-spectrum beta-lactamase-producing and carbapenem-resistant bacteria is undermining the efficacy of beta-lactam antibiotics, largely due to empirical antibiotic use without precise pathogen identification (Santella et al., 2021). Hospital-acquired pneumonia (HAP) is particularly common in intensive care units (ICUs), affecting 15%−20% of hospitalized patients in the United States, and represents a leading cause of mortality among ICU-associated infections (Georgakopoulou et al., 2023). These pathogens have been recognized by the WHO as a critical threat to public health, causing infections such as pneumonia, bacteraemia, septicaemia, and urinary tract infections (UTIs) (Wang et al., 2023; Novick, 2021; Seccareccia et al., 2015; Caulton et al., 2024).

Predatory bacteria have attracted increasing attention in the current era of antimicrobial resistance. Their effectiveness is determined by their ability to kill or incapacitate target bacteria (Novick, 2021). Predatory mechanisms can be broadly classified into three modes: endobiotic predation, in which the predator inhabits and reproduces within its host; epibiotic predation, which occurs extracellularly; and transbiotic predation, involving the release of lytic proteins that degrade prey cells (Novick, 2021; Seccareccia et al., 2015). *Bdellovibrio bacteriovorus* represents a classical endobiotic predator, whereas *Micavibrio aeruginosavorus* is an epibiotic prototype. Transbiotic predators are generally not of direct clinical interest, although some of their enzymatic products have therapeutic relevance (Novick, 2021).

## 2 Methodology

To explore the therapeutic approach of *Bdellovibrio bacteriovorus* against multidrug-resistant respiratory infections, a thorough literature search was performed. The three main databases (Google Scholar, PubMed, Scopus) were pressed into service to gather all the relevant information. A conscientious approach was employed, focusing on studies that met specific inclusion criteria. The criteria included diverse study designs, including trials and real-world studies with a main focus on mechanisms, safety, and clinical application of “*Bdellovibrio bacteriovorus*.”

To ensure the cohesion and integrity of the narrative review, studies conducted in languages other than English were excluded. This ruling was aimed at sustaining a clear and focused narrative. The search strings used during the literature search targeted areas of interest. Keywords such as “*Bdellovibrio bacteriovorus*” OR “Predatory bacteria” AND “Multidrug resistant bacteria” OR “Respiratory infections” OR “Biological antimicrobial strategy” were included to collect appropriate studies. The literature search encompassed studies published until January 2025. The initial database search yielded approximately 420 records. After removal of duplicates, 186 studies were screened based on titles and abstracts. Following full-text evaluation, 92 articles were included in this narrative review based on relevance to predatory mechanisms, antimicrobial activity, safety, and therapeutic application. As this was a narrative review, no formal risk-of-bias or quality assessment tool was applied.

## 3 About *Bdellovibrio bacteriovorus*

Since its discovery in 1963 by Stolp and Starr *Bdellovibrio bacteriovorus* has gained attention because of increased antibiotic resistance, positioning it as a potential probiotic and antibiotic agent against bacteria (Du Toit, 2024). *B. bacteriovorus* derives its name from the Latin “bdella,” meaning leech-like, and “vibrio,” signifying its comma shape. This Deltaproteobacteria, measuring approximately 0.3–0.5 μm by 0.5–1.4 μm, is a Gram-negative, monotrichous, ubiquitous, obligate aerobic bacterium having a unique ability to prey on other Gram-negative bacteria (Caulton et al., 2024; Du Toit, 2024; Cavallo et al., 2021).

This ability of *B. bacteriovorus* to feast upon other Gram-negative bacteria is due to its host-dependent reproductive mechanism. Briefly, the mechanism initially involves the bacteria using propulsion through their flagellum and reversibly binding with the external membrane of the potential host for a short “recognition period”; however, after which it becomes irreversibly attached (Du Toit, 2024; Lambert et al., 2009; Lai et al., 2023; Lambert et al., 2016). This propulsion from its flagellum helps it to reach speeds of more than 160 μm/s, roughly a distance of 160 body lengths per second (Lambert et al., 2009). Then, upon encountering suitable prey, invasion of the prey's outer membrane and the colonization of its periplasm begin. A characteristic round-shaped structure forms between the prey and predator called a bdelloplast, which marks the start of the growth phase (Lambert et al., 2009; Lai et al., 2023). In bdelloplast, the prey's peptidoglycan is altered to prevent bursting and instead provides space for the predator's growth. The prey's cellular components are broken down into monomers, which are then absorbed and utilized to fuel the *Bdellovibrio's* growth and replication (Lambert et al., 2016). After that, the predator then lives off the intracellular components of the victim, and when the nutrients are exhausted, the progeny predators septate while the host undergoes lysis.

**Figure 1:**
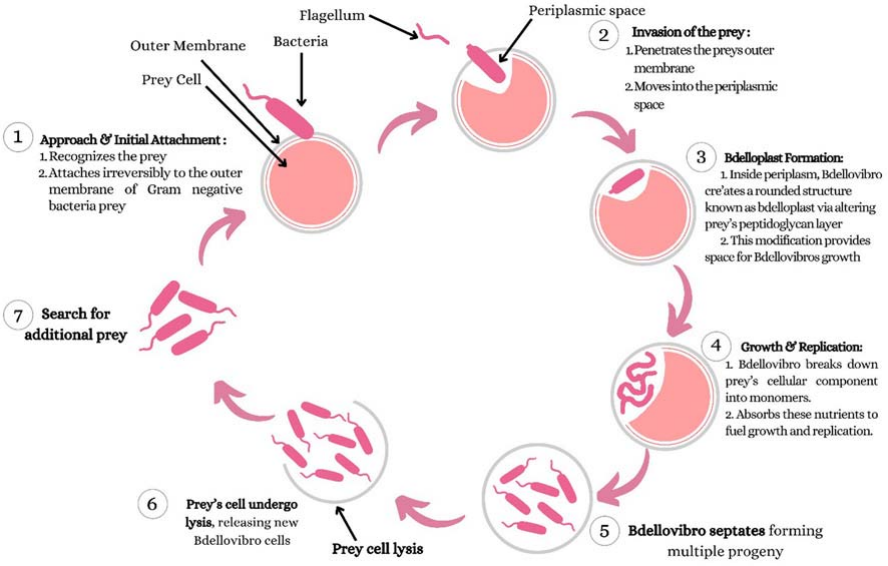
Predatory life cycle of *Bdellovibrio bacteriovorus* against Gram-negative bacteria. The figure illustrates the sequential stages of *Bdellovibrio bacteriovorus* predation. **(1)** Approach and initial attachment: motile *B. bacteriovorus* locates Gram-negative prey using chemotaxis and reversibly attaches to the prey's outer membrane before irreversible attachment occurs. **(2)** Invasion of the periplasmic space: the predator penetrates the prey's outer membrane and enters the periplasm without disrupting the cytoplasmic membrane. **(3)** Bdelloplast formation: the prey cell is rounded into a stable structure termed the bdelloplast through modification of the peptidoglycan layer, providing a protected niche for predator growth. **(4)** Growth and replication: *B. bacteriovorus* degrades prey cellular components into utilizable monomers and elongates within the bdelloplast. **(5)** Septation: the elongated predator divides synchronously into multiple progeny cells. **(6)** Prey cell lysis: enzymatic degradation of the prey cell wall releases newly formed *B. bacteriovorus* cells. **(7)** Search for new prey: progeny cells regain motility and seek additional Gram-negative bacteria, perpetuating the predatory cycle. This life cycle underpins the potential of *B. bacteriovorus* as a biological antimicrobial agent.

Furthermore, this sequence perpetuates until the prey is reduced to a small number. Interestingly, suppose the first invasion did not provide sufficient nutrients for a complete replication. In that case, a *B. bacteriovorus* cell may leave the first bdelloplast and complete its life cycle in a second prey (Makowski et al., 2019). This cycle of *B. bacteriovorus* is further illustrated in Figure 1. Aside from the predatory life cycle, *B. bacteriovorus* also displays a host-independent reproductive mechanism, characterized by a saprophytic, non-virulent phase when a suitable host for the predator is absent (Lambert et al., 2009).

### 3.1 Molecular determinants of predation

Predation by *B. bacteriovorus* involves coordinated molecular mechanisms governing prey recognition, invasion, and lysis. Initial attachment is mediated by chimeric fiber proteins enabling recognition of diverse outer membrane structures (Caulton et al., 2024). Recent genomic studies reveal modular fiber architectures that enhance prey adaptability and host range (Santella et al., 2021). Following attachment, *B. bacteriovorus* secretes lytic enzymes, including peptidoglycan hydrolases and proteases, facilitating bdelloplast formation and controlled prey remodeling (Duan et al., 2020; GBD 2015 LRI Collaborators, 2017). These processes are tightly regulated by signaling pathways that coordinate the transition between attack and growth phases (Georgakopoulou et al., 2023). Together, these molecular adaptations underscore the evolutionary specialization of *B. bacteriovorus* as a predator rather than a conventional antimicrobial agent.

## 4 *Bdellovibrio* against Gram-negatives

*Bdellovibrio bacteriovorus* is a well-known *Gram-negative* hunter. Studies have shown *B. bacteriovorus* targeting the pathogenic and opportunistic bacteria belonging to the genera *Pseudomonas, Acinetobacter*, and *Klebsiella*, amongst many others, with the predators maintaining their ability to prey on the host cells despite the MDR (multidrug resistant) status (Sun et al., 2017).

In a study conducted by Shatzkes et al. (2016), Sprague-Dawley (SD) rats were exposed to a sublethal dose of *K. pneumoniae* via intranasal inoculation. *K. pneumoniae* is an opportunistic pathogen responsible for healthcare-associated infections, mostly targeting immunocompromised individuals or patients with concurrent infections (Arato et al., 2021). Because *Gram-negative* bacteria do not find good growth conditions on the human skin (Myles et al., 2016), *Klebsiella* spp. are rarely found there and are regarded simply as transient members of the flora (Quintelas et al., 2024). Its virulence factors, which include capsule polysaccharide along with its high genetic variability, make it particularly resistant to antibiotics (Arato et al., 2021). It has been recognized by the WHO as a critical threat to public health, causing infections such as pneumonia, bacteraemia, septicaemia, and UTIs (Arato et al., 2021; World Health Organization, 2017b). Naturally equipped with beta-lactamases and carbapenemases, *K. pneumoniae* is responsible for high mortality rates (40%−50%), particularly in immunocompromised or critically ill individuals (Martin and Bachman, 2018).

Regardless, in an *in vivo* study, after these rats were exposed and inoculated with *K. pneumoniae* in their lungs, few of these rats received an additional inoculation of 4.6 × 10^8^ Plaque Forming Units (PFU)/rat of *B. bacteriovorus*. In contrast, others received phosphate-buffered saline (PBS) as a control group, administered over 24 h post-infection from *K. pneumoniae*. The predatory bacteria and control were also administered via intranasal inoculation at intervals of 30 min, 6, 12, and 18 h before the animals were sacrificed, and their organs were harvested at 24 h post-inoculation (Shatzkes et al., 2016).

After the homogenizing and plating of the lung tissues in MacConkey agar, it was observed that in *K. pneumoniae* inoculated lungs, 75.0 percent of the rats initially infected by *K. pneumoniae* and further treated by the PBS control showed *K pneumoniae* state. On the other hand, *K. pneumoniae* colonies were isolated in the lungs of only 58.3 percent of *K. pneumoniae* inoculated rats and treated with *B. bacteriovorus*. Furthermore, 83.3% of rats treated with *B. bacteriovorus* exhibited reductions in copy numbers of *K. pneumoniae* by more than 3.0 log_10_ compared to the mean of the results from PBS-treated rats, indicating the efficiency of *B. bacteriovorus* in reducing bacterial burden in rat lungs (Iebba et al., 2014). It demonstrated that *B. bacteriovorus* efficiently killed bacteria in the respiratory tract of rats.

*Pseudomonas aeruginosa*, a Gram-negative rod notorious for its multidrug resistance, is a common opportunistic pathogen in the hospital setting. Causing a large number of nosocomial infections, particularly in immunocompromised individuals, the WHO has cited it as one of the greatest threats to humans concerning AMR. The abundance and diversity of virulence factors, as well as the secretion of certain products like exopolysaccharides, proteases, and siderophores, make this species particularly resistant to antibiotic therapy (Liao et al., 2022). It is also one of the bacteria most commonly affecting patients with cystic fibrosis (Iebba et al., 2014). It is particularly resistant to the innate immune effectors and to antibiotics due to the expression of certain specific virulence factors, including antioxidants and exopolysaccharides, and by also acquiring genetic mutations to adapt during chronic infections (Malhotra et al., 2019). Biofilm formation also helps these bacteria to survive in chronic infections (Iebba et al., 2014).

Biofilms are intricate communities of matrix-enclosed, surface-associated microbial cells and noncellular components and are a common feature of many bacteria. The extracellular matrix acts as a scavenging network to capture and accumulate necessary minerals or nutrients from the external environment (Costerton et al., 1994). It can also offer some kind of immunity to environmental hazards. In addition, the presence of biofilm significantly reduces the efficacy of the antimicrobial and the immune system of the host. For this, several mechanisms have been proposed; one such mechanism for the reduced effectiveness of antimicrobial agents is the possibility that the antimicrobial agents may be entrenched into the biofilm matrix, where they can undergo chelation due to deactivation enzymes. There is also a possibility that it could be a consequence of high bacterial density of the biofilm's microcolonies that leads to waste buildup and microenvironment change, which may compromise antimicrobial action deep within the biofilm. Another theory is the presence of slow or non-growing bacteria, called “persister cells,” deep in the biofilm. Persister cells are more durable, tough, and may survive the initial antimicrobial assault; however, upon discontinuation of the therapy, they would be able to reconstitute the biofilm (Abrantes and da Rocha Nogueira, 2022).

When *P. aeruginosa* was isolated from the sputa of two cystic fibrosis patients, it was observed that *B. bacteriovorus* effectively decreased the population of this pathogenic bacterium. Furthermore, when 24-h-old biofilms of *P. aeruginosa* strains were subjected to 24 h of *B. bacteriovorus* predation, there was a phenomenal 76% reduction in *P. aeruginosa* biofilm biomass (Iebba et al., 2014).

Further studies in the literature also report similar bacterivorous effectiveness against many such biofilms (Monnappa et al., 2014; Vatansever et al., 2023). This effectiveness could be attributed to *B. bacteriovorus*' unique ability to penetrate deeply into prey biofilms, efficiently eradicating them—a distinguishing feature not shared by other biological agents such as bacteriophages and protists, which are frequently observed to be only restricted to the surface of the biofilm (Sun et al., 2017). Additionally, it is also suspected that biofilms might provide favorable conditions for the survival of *B. bacteriovorus*. Such claims are made because these predators have been detected lurking in natural marine biofilms, even though they are not consistently found in the surrounding water (Negus et al., 2017). This distribution suggests that *B. bacteriovorus* prefers living in biofilms, which could be attributed to higher prey densities found within these structures. As prey density increases, so do opportunities for *bdellovibrio* infection, growth, and population stability. Besides this, biofilms may offer a degree of protection against environmental forces for predatory bacteria as well, further enhancing their survival prospects (Wucher et al., 2020). So, ironically, the very thing bacteria produce for protection in incidences of environmental stress is being used against them.

## 5 *Bdellovibrio* against gram-positives

Although the majority of respiratory infections are associated with *Gram-negative* infections, gram-positive infections are also suspected to cause a significant number of respiratory tract infections (Shatzkes et al., 2015). In a detailed study at an Italian hospital from 2015 to 2019, researchers analyzed 7,038 sputum and bronchial aspirate specimens isolated from patients with lower respiratory tract infections, where the infections were confirmed via microscopic examinations. They found that under the *Gram-positive* category, *Staphylococcus aureus* was the most common bacterium causing lower respiratory tract infections (Shatzkes et al., 2015).

*Staphylococcus aureus* is a pathogen significant in clinical settings that has a plethora of infections associated with it, ranging from minor infections of the skin to severe tissue and systemic infections such as sepsis (blood poisoning). It is a frequently known cause of nosocomial infections because it has a high degree of antibiotic resistance. In addition to that, since the early 1960s, the emerging epidemic of community-acquired methicillin-resistant *S. aureus*, plus the overall very severe nature of *Staphylococcus* infections, has resulted in frequent misuse of anti-staphylococcal antibiotics, only contributing to the ever-increasing resistance rates (Ahmad-Mansour et al., 2021). While *B. bacteriovorus* has traditionally been associated with predation on Gram-negative bacteria, emerging research suggests its potential efficacy against Gram-positive counterparts as well (Pantanella et al., 2018). The other common species in cystic fibrosis patients is *S. aureus*. Hence, when Iebba et al. (2014) collected sputa from cystic fibrosis patients, they also experimented with the efficacy of *B. bacteriovorus* against *S. aureus*. Despite prior literature continuously suggesting that *bdellovibrio* does not prey on Gram-positive bacteria, under higher Field-Emission Scanning Electron Microscopy (FESEM), the authors found that *B. bacteriovorus* preys on *S. aureus* in an epibiotic manner, a completely different mechanism from that observed with Gram-negative bacteria.

Unlike the periplasmic invasion mechanism observed in Gram-negative prey, interactions with Gram-positive bacteria occur through epibiotic attachment without intracellular replication. This mechanism appears to be strain-dependent and may have important implications for polymicrobial respiratory infections involving both Gram-negative and Gram-positive pathogens.

Regardless of the particular prey preference *bdellovibrio* displays, as already mentioned, *B. bacteriovorus* could be used against *S. aureus* biofilms. Furthermore, even if *B. bacteriovorus* does not directly prey on *Staphylococcus aureus*, they are reported to reduce virulence and invasion of *Staphylococcus aureus* into human epithelial cells. On exposing an *S. aureus* culture to *B. bacteriovorus* at 2 h, the authors observed a nearly 5-fold reduction in the number of invasion events as compared to that of the untreated bacterial culture.

On further inspection via a sodium dodecyl sulfate–polyacrylamide gel electrophoresis (SDS-PAGE) analysis, the study authors found that the treatment with predatory bacteria has significantly reduced *S. aureus* surface proteins; such proteins are responsible for essential virulent functions of this pathogen, aside from other important functions, for example, biofilm formation (Monnappa et al., 2014). Importantly, these epibiotic interactions do not represent true predation in the classical sense and should not be interpreted as equivalent to periplasmic invasion observed in Gram-negative prey.

Importantly, this epibiotic interaction does not constitute true predation, as Gram-positive bacteria lack an outer membrane and periplasmic space required for bdelloplast formation and intracellular replication. Consequently, *B. bacteriovorus* is unable to complete its canonical life cycle within Gram-positive hosts, resulting in inconsistent killing that is likely strain-dependent and environmentally influenced rather than a reproducible predatory mechanism.

## 6 Safety of *Bdellovibrio bacteriovorus*

Surprisingly, despite the significant efficacy of *B. bacteriovorus*, literature reports no adverse effect associated with its use. When Shatzkes et al. (2016) investigated the safety of *B. bacteriovorus* administration, they noted that all predatory bacteria-infected rats did not show any symptoms of illness or being unwell. Lung tissue histology analysis at 24- and 48-h post-inoculation showed no abnormal pathology with *B. bacteriovorus*, and at 10 days post-inoculation of *B. bacteriovorus*, the lungs of rats did not have any predatory bacteria. Inflammatory proteins analyzed by ELISA demonstrated an initial increase of 59.3-fold and 3.7-fold in tumor necrosis factor-alpha (TNF-alpha) and KC/GRO (CXCL1), respectively, evaluated 1-h post-inoculation in pathogen-positive rats inoculated with *B. bacteriovorus*. Nevertheless, these elevations were not maintained over time because by 24 h following inoculation, the concentrations of TNF-alpha as well as KC/GRO reverted to baseline levels. Similar results were established regarding the IL-6 (interleukin-6) and IL-13 levels. The authors followed up to report that none of the remaining inflammatory cytokines showed increases above 3.0-fold at any time point in any group exposed to predatory bacteria, indicating the safety of these bacteria (Shatzkes et al., 2015). The same effects of the *B. bacteriovorus* were identified in another research, where intranasal and intravenous infections were employed (Shatzkes et al., 2015). There was no impairment in the viability of the mouse following intranasal or intravenous inoculation. Though there was a modest upsurge in the introduction of such predators in the respiratory tract, in mice, a modest inflammatory response was observed after 1 h of exposure, but it was not observed for 24 h, as shown by RT-qPCR and ELISA (Shatzkes et al., 2015). With intravenous injection, there was an elevation in IL-6 in the spleen and kidney, TNF in the liver, and CXCL-1/KC discernible in the blood at 3 h post-exposure, and decreased to the baseline level at 18 h after inoculation (Saralegui et al., 2022; Shatzkes et al., 2015).

On top of that, the predators were eliminated in the host fast and effectively, as identified by qPCR. No pathologic changes caused by the bacteria were also seen in the histological analysis of the tissues (Shatzkes et al., 2015). Although irrelevant to lung infections, further *in vivo* studies also confirm their safety (Ahmad-Mansour et al., 2021; Shatzkes et al., 2017). However, studies involving *bacteriovorus* usage for gut infections do suggest a change in intestinal flora.

Although short-term animal studies demonstrate a favorable safety profile, no long-term toxicity studies or human clinical trials evaluating *B. bacteriovorus* are currently available. Additionally, while preliminary gut microbiome studies suggest minimal disruption of commensal flora, the long-term impact on the respiratory microbiome remains unknown and warrants further investigation.

## 7 Immune response to *Bdellovibrio bacteriovorus*

The lack of an immune reaction to these predatory bacteria might be due in part to changes in a substance called lipopolysaccharide (LPS) (Madhusoodanan, 2019). The antagonistic activity of an immune response is primarily attributed to the presence of negatively charged phosphate residues of the LPS on the surface of Gram-negative bacteria, which have a strong binding effect on Toll-like receptors of immune cells. Neutrally charged LPS appears to be expressed by *B. bacteriovorus*, triggering *in vitro* only a very weak inflammatory response (Madhusoodanan, 2019).

## 8 Methods of application and synergistic strategies

The usage of *bacteriovorus*, however, should not be thought of as being perfect. This bacterium has some limitations on what it can do. For example, although *bdellovibrio* prey on *Gram-negative* bacteria, they never wholly eradicate their prey, despite having excessive predator-to-prey ratios. As the prey population dwindles, the remaining victim bacteria display transient resistance and, upon further investigation, are seen to be a plastic response under stress caused by the predator rather than a mutational event. This resistance leads to the prey population rebounding *in vitro* (Hobley et al., 2020). One could say that the *bacteriovorus* reduces the burden of pathogens enough that the immune system can now handle it easily. This hypothesis is supported by limited experimental evidence, including a single study in which *bdellovibrio* treatment was associated with reduced *Shigella* burdens in both immunocompromised and immunocompetent zebrafish larvae. However, survival was markedly higher in immunocompetent larvae. This might imply that this stage of predation-killing has the potential to suppress the pathogen so that it can be cleared by the innate immunity of the vertebrate. If one might want to eradicate all the pathogenic prey bacteria, the predators can be alternated with another agent to prevent the gram-negative bacteria from developing resistance. As the predators have a naturally occurring resistance to β-lactam antibiotics (Kuru et al., 2017), the approach of combining *bdellovibrios* with penicillin therapy might be practicable. Unlike in phage therapy, another approach being researched for therapeutic use, where a single point mutation can eradicate the therapeutic interaction, *Bdellovibrio* do not face these limitations, as they have a broad prey range, giving them much more potential to be used as an alternative to traditional antibiotics.

**Figure 2:**
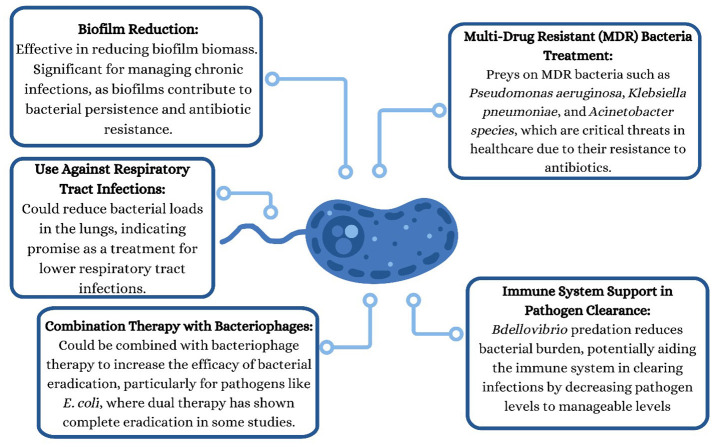
Therapeutic applications of *Bdellovibrio bacteriovorus* in antimicrobial strategies. *Bdellovibrio bacteriovorus* demonstrates multiple clinically relevant applications, including: (i) reduction of biofilm biomass, enhancing susceptibility of biofilm-associated pathogens to immune clearance and antimicrobials (Vatansever et al., 2023); (ii) treatment of multidrug-resistant (MDR) Gram-negative bacteria, including *Pseudomonas aeruginosa, Klebsiella pneumoniae*, and *Acinetobacter* species (Sun et al., 2017); (iii) adjunctive use in respiratory tract infections, where intranasal administration reduces pulmonary bacterial burden *in vivo* (Shatzkes et al., 2016); (iv) combination therapy with bacteriophages, resulting in synergistic and complete eradication of bacterial populations in experimental models (Hobley et al., 2020); and (v) immune system support through pathogen load reduction, facilitating host immune clearance without sustained inflammatory responses (Shatzkes et al., 2015). These applications highlight the potential role of *B. bacteriovorus* as a living antimicrobial alternative or adjunct to conventional antibiotic therapies.

Furthermore, interestingly, from the point of view of the prey, the adaptations acquired due to the increased resistance to *B. bacteriovorus* predation make the *Gram-negative* bacteria increasingly susceptible to attacks by bacteriophages due to the presence of specific phage receptors in the cell envelope of the bacteria. So, uniting Bdellovibrio with phage therapy for therapeutic use might also be feasible. In fact, in one set of experiments, using this particular strategy on a colony of *E. coli*, the pair reduced prey numbers by a much larger magnitude than either of the predators independently of each other did. The duo caused a complete eradication of the prey *E. coli* (reduction below detectable levels of < 10 cells/ml) (Hobley et al., 2020). Similarly, *in vitro* studies have shown that *B. bacteriovorus* may exhibit enhanced antibacterial activity when used in combination with probiotic or commensal bacteria, including *Pseudomonas* fluorescens and Lactobacillus acidophilus, supporting the feasibility of multi-agent biological strategies against Gram-negative pathogens (Aksono et al., 2025). One major limitation of B. *bacteriovorus* is the presence of paracrystalline protein surface layers (S-layers) on certain prey bacteria. Koval and Hynes demonstrated that prey bacteria possessing an intact S-layer are resistant to *Bdellovibrio* predation, rendering the predator ineffective against pathogens with a complete S-layer. However, it is worth mentioning that even small kinks in this bacterial armor make the prey bacteria susceptible to *B. bacteriovorus* again (Cavallo et al., 2021). The therapeutic applications of *B. bacteriovorus* are further explained in Figure 2.

## 9 Limitations and future directions

Despite its promise, *B. bacteriovorus* exhibits several limitations. Complete eradication of prey populations is rarely achieved, even at high predator-to-prey ratios. Surviving bacteria often display transient, non-mutational resistance driven by stress-induced phenotypic plasticity. Additionally, prey expressing intact S-layers are partially protected from predation. Translational challenges include ensuring predator stability in the respiratory tract, optimizing delivery methods such as nebulization, and navigating regulatory frameworks for live biotherapeutics. Future research should prioritize long-term safety studies, microbiome impact assessments, and controlled human trials. Collectively, these limitations emphasize that *B. bacteriovorus* should currently be viewed as a complementary or adjunctive strategy rather than a standalone replacement for conventional antimicrobial therapies.

## 10 Conclusion

When penicillin was discovered, history was made. Although predatory bacteria may have some weaknesses and not be the “Wonder Drug” as before, they may provide valuable aid in our constant fight against bacteria. With the ever-looming threat of forever-increasing AMR and our continuous battle with multidrug-resistant strains of bacteria, it is of utmost importance to explore alternatives to traditional antibiotics. The novel approach of introducing predatory bacteria, such as *B. bacteriovorus*, therapeutically to fight against infections, especially against multidrug-resistant respiratory tract infections, might potentially be viable in the future. It might open new paths in medicine to fight against such prevalent infections. Although more research needs to be done, *in vivo* studies showed promise, and no major side effects of the administration of such bacteria in the body were found. In Eastern Europe, bacteriophages are already serving as an antimicrobial treatment (Kakasis and Panitsa, 2019). Such efficacy and safety support cautious progression toward regulated human studies, potentially beginning with compassionate-use frameworks.
